# Effects of Resveratrol on Crosstalk between Canonical Β-Catenin/Wnt and FOXO Pathways in Coronary Artery Disease Patients with Metabolic Syndrome: A Case Control Study

**Published:** 2016

**Authors:** Mehrnoosh Shanaki, Arash Hossein-nezhad, Reza Meshkani, Maani Beigy, Mahmoud Shirzad, Parvin Pasalar, Taghi Golmohammadi

**Affiliations:** a *Department of Biochemistry, School of Medicine, Tehran University of Medical Sciences, Tehran, Iran.*; b *Department of Medical Laboratory Sciences, School of Allied Medical Sciences, Shahid Beheshti University of Medical Sciences, Tehran, Iran. *; c *Osteoporosis Research Center, Endocrinology and Metabolism Clinical Sciences Institute, Tehran University of Medical Sciences, Tehran, Iran,*; d *Department of Medicine, Section of Endocrinology, Nutrition, and Diabetes, Vitamin D, Skin, and Bone Research Laboratory, Boston University Medical Center, Boston, MA 02118, USA,*; e *Students’ Scientific Research Center, Tehran University of Medical Sciences, Tehran, Iran. *; f *Cardiovascular Surgery Department, Tehran Heart Center ,Tehran University of Medical Sciences, Tehran, Iran.*

**Keywords:** Coronary artery disease, Metabolic syndrome, Resveratrol, β-catenin, Wnt signaling, FOXO, MnSOD

## Abstract

Coronary artery disease (CAD) is the major cause of mortality and morbidity worldwide. The aim of this study was to explore the effect of resveratrol (RES) on Canonical β-catenin/Wnt and forkhead box O (FOXO) pathways in CAD patients.

We performed this study on 10 metabolic syndrome patients with three-vessel CAD and 10 sex-aged matched healthy subjects. The effects of RES on β-Catenin, manganese superoxide dismutase (MnSOD), and peroxisome proliferator-activated receptor delta (PPAR-δ) expression were evaluated in peripheral blood mononuclear cells (PBMCs) of participants.

RES could increase the MnSOD expression in CAD patients (38%, p < 0.0001). After RES treatment, the MnSOD expression of patients is still non-significantly lower than controls. In both blank and RES treatments, a significant positive correlation between β-catenin and MnSOD mRNA expressions was found in controls, whereas no correlation between these gene expressions was found in untreated PBMCs of CAD patients. However, RES could modestly improve this pathway in CAD. RES could increase the MnSOD activity in healthy and CAD subjects (p = 0.051 and p = 0.009, respectively). Furthermore, in both blank and RES treatments, the significant correlation was found between total β-catenin protein and the MnSOD activity in PBMCs of the controls but not in patients.

The cross-talk between β-catenin/Wnt and FOXO pathways was impaired in PBMCs of CAD patients. RES treatment could lead to a modest increase in the MnSOD activity independent of β-catenin/FOXO pathway. Despite a modest improvement in the β-catenin/FOXO pathway after RES treatment, this pathway was not completely repaired in CAD patients.

## Introduction

Metabolic syndrome is a constellation of cardiovascular and metabolic risk factors including obesity, insulin resistance, hypertension and dyslipidemia. Coronary artery disease (CAD) is considerably linked with these risk factors (1, 2). Oxidative stress has a major role in development of atherosclerosis that is believed as the most common pathologic process underlying CAD which is the leading cause of mortality and morbidity worldwide (1, 3, 4). In this condition, the imbalanced production of reactive oxygen species could trigger lipid and protein oxidation in the vascular wall (3). 

In order to reduce the deleterious effects of oxidative stress, several antioxidant protective networks and signaling pathways are operative in cells. The up-regulated gene-expression of free radical scavenging enzymes such as manganese superoxide dismutase (MnSOD) by members of the forkhead box O (FOXO) transcription factors is considered to be one of the paramount cell defensive mechanisms against oxidative damage (5, 6). It is now well recognized that β-catenin binds to FOXOs during oxidative stress and acts as the pivotal mediator in canonical Wnt signaling, so that it translocates to the nucleus and interacts with the family of transcription factors T-cell factor/lymphoid enhancer factor (TCF/LEF), to regulate the expression of Wnt target genes (6, 7). Recent evidence suggested that the canonical Wnt signaling plays a profound role in regulation of lipid metabolism and glucose homeostasis (8). Peroxisome proliferator-activated receptor delta (PPAR-δ) is one of the Wnt target genes which is believed to be operative in cardiometabolic protection (9). Interestingly, it has been demonstrated that impaired Wnt signaling pathway is contributed to inflammation, foam cell formation, and endothelial dysfunction which are recognized as atherosclerosis pathogenic factors (10).

Resveratrol (RES) (3, 4´, 5 trihydroxystilbene), a natural polyphenol with antioxidant effects can be found in red grapes and its processed drinks (*e.g*. red wine), peanuts, pomegranates and mulberries (11-13). Increasing body of evidence suggest a protective role for RES against CAD (14-16), however the underlying mechanisms still remain to be elucidated. Moreover, the dysfunctions of Wnt signaling pathway has been comprehensively studied in pathogenesis of malignancies, while based on literature it has not yet been investigated in CAD patients. Thus, we conducted this study in order to explore the alterations of β-catenin/Wnt and FOXO signaling pathways by investigating their selected target genes (MnSOD and PPAR- δ) in peripheral blood mononuclear cells (PBMCs) of patients with CAD. In addition, in view of the plausible role of β-catenin/FOXO pathway in regulation of oxidative stress which is involved in the pathogenesis of CAD, it seems that anti-oxidants could be considered as promising agents in preventing the CAD related oxidative stress. In the current study, we therefore sought to investigate RES effects on cross-talk between β-catenin/Wnt and FOXO signaling pathways in CAD patients which has chronic oxidative stress.

## Experimental

*Study design and patient selection*

This case control study was conducted on 10 metabolic syndrome patients with three vessel CAD and 10 healthy subjects. Patients and healthy subjects were matched regarding gender (men) and age (40-55). The National Cholesterol Education Program Adult Treatment Panel III (NCEP ATP III) criteria was used to diagnose the metabolic syndrome (17) as presenting at least three of the following components: 1) elevated waist circumference (102 cm in men and 88 cm in women), 2) hypertriglyceridemia (≥ 150 mg/dL), low high density lipoprotein (HDL) cholesterol level (≤ 40 mg/dL), high blood pressure (systolic blood pressure ≥ 130 mmHg and/or diastolic blood pressure ≥85 mmHg and/or pharmacological treatment), and elevated fasting glucose (≥110 mg/dL) and/or pharmacological treatment. 

All patients underwent coronary angiography to assess the severity of CAD at Tehran Heart Center (Affiliated by Tehran University of Medical Sciences). The severity of CAD was defined in terms of number of involved vessels with significant stenosis (> 50 % narrowing in coronary arteries). The exclusion criteria were established as having malignancy, myocardial infarction, unstable angina, previous coronary intervention, inflammatory diseases and other known chronic diseases. The subjects receiving antioxidant therapy or vitamin supplements in the previous 12 months, and also smokers were not included in the study. Five patients were under treatment with cholesterol lowering and anti-hypertensive medications. The acetylsalicylic acid usage was stopped one week prior to coronary artery bypass graft (CABG) surgery. Healthy subjects were selected from the healthy population with normal electrocardiographic findings. This group had normal lipid profile with no history of diabetes, hypertension, endocrine disorders, and cancer. The study was approved by Tehran University of Medical Sciences (TUMS) Ethics Committee, and written informed consent was obtained from all subjects.

Participants’ information was obtained by questionnaires on personal data and clinical measurements such as age, gender, drug consumption during the past months, and medical or family history of diabetes. Systolic and diastolic blood pressure was measured twice in the right arm of the subjects who had been resting for at least 10 min in a comfortable position (Omron, M6 Comfort HEM-7321-E, Japan). Body mass index (BMI) was calculated as weight in kilograms divided by the height in meters squared. The waist circumference was taken at the midpoint between the iliac crest and the lower rib margin.

*Blood sampling*

Venous Blood samples (25 cc) were obtained from participants after overnight fasting between 7:00 and 9:00 am. Then the samples were divided into two aliquots, in clot activator tube (5cc) and heparin-treated vacutainer (20 cc) in order to biochemical analyses and PBMC isolation, respectively. In addition, 1 cc blood was used for plasma ferric reducing antioxidant power (FRAP) assay. Serum and plasma samples were obtained after centrifuge at 300 g for 15 min at 4 ˚C, and were stored at -80 ˚C for further experiments. 

*Laboratory measurements*

Serum fasting levels of glucose (FBS), HDL cholesterol, low density lipoprotein (LDL) cholesterol, total cholesterol (TC) and triglyceride (TG) were measured by Pars Azmoon kits (Iran) using auto-analyzing system (Autoanalyzer, Hitachi 917 Ltd, Tokyo, Japan). 

*PBMC separation*

Human PBMCs were separated by the Ficoll-Hypaque (lympholyte-H) (Cedarlane, Canada) gradient centrifugation method from heparinized blood samples. Freshly isolated PBMC were re-suspended in RPMI-1640 medium (Gibco, USA) containing 10% heat-inactivated fetal bovine serum (Gibco, USA), and 1% penicillin/streptomycin solution (Gibco, USA). The numbers of viable PBMCs were determined with trypan blue exclusion method. PBMCs were plated at density of 2 × 10^6^ cells/well in 12-well plates, in order to further experiments. RES (Sigma-Aldrich, St. Louis, MO, USA) was dissolved in dimethyl sulfoxide (DMSO) (Sigma-Aldrich, St. Louis, MO, USA), in which DMSO final concentration in culture medium was less than 0.025%. Blank group (untreated cells) received the same amount of DMSO.

*Evaluation of cell viability under RES treatment*

PBMCs (25 × 10^4^/well) were seeded in 96-well plates and underwent overnight incubation in humidified atmosphere at 37 ˚C temperature with 5% CO_2_, then the medium was removed by centrifugation at 300 g for 15 min and replaced with a fresh medium containing two concentrations of RES (50 and 100 µM) prepared in FBS-free RPMI-1640. After 12 h and 24 h incubation, the cells were rinsed three times with Phosphate-buffered saline tablets (Gibco, USA), and then 5 mg/mL 3-(4, 5-dimethylthiazol-2-yl)-2, 5 diphenyltetrazolium bromide (MTT) (Sigma-Aldrich, St. Louis, MO, USA) was added into the wells. After 4 h the medium was removed and the blue formazan crystals were solubilized with 100 μL of DMSO. Optical density was recorded at 540 nm. Cell viability assessment was performed based on the reduction of MTT by mitochondrial dehydrogenase of viable cells. The percentage of viable cells was calculated relative to untreated cells. 

*Quantitative Real-time PCR*

For RNA extraction, PBMCs (2 × 10^6^ cells/well) were seeded in 12-well plates. RNA extraction was performed using Total RNA Extraction Miniprep kit (VIOGENE, Taiwan) according to the manufacturer’s protocol. The RNA concentration was determined by measuring the 260/280 nm absorbance ratio and its quality was evaluated using agarose gel electrophoresis. First-strand cDNA synthesis was carried out using 1 µg RNA with cDNA Revert Aid First Strand cDNA Synthesis Kit (Thermo scientific, Fermentas, USA). The resulting cDNA were amplified using SYBR Green PCR Master Mix (Takara, Japan) by Rotor Gene real-time thermocycler (QIAGEN, Hilden, Germany) according to the manufacturer›s protocol. MnSOD, PPAR-δ, β-catenin and β-actin primers were purchased from QIAGEN (Hilden, Germany). All gene-expression data were normalized to β-actin (ΔCT). The standard curves were generated from the pooled cDNA of the assayed samples.

*MnSOD enzyme activity and total β-catenin protein measurement*

MnSOD activity was measured by the SOD activity kit (ADI-900-157; Enzo Life Sciences; USA), through colorimetric-based assay at 450 nm. Briefly, MnSOD prevents the reduction of WST-1 (water soluble tetrazolium salt) to WST-1 formazan by neutralizing superoxide anions radicals which are generated from the xanthine oxidase function (in the presence of xanthine and O2). MnSOD was detected from total SOD by administering potassium cyanide (KCN) 1 mM which inhibits both Cu/Zn-SOD and extracellular SOD. Total β-catenin protein (non-phosphorylated active form plus phosphorylated inactive form) was measured by enzyme immunoassay (EIA) according to the protocol described in the kit (ADI-900-135; Enzo Life Sciences; USA). Determination of PBMC numbers for enzyme activity and protein level measurement were performed according to kit manufacture protocol. The cells were harvested through trypsinization (for MnSOD enzyme activity) and scraping (for total β-catenin protein). Protein estimation of each sample was performed using the method of Bradford (18).

*FRAP assay*

Total antioxidant capacity was determined by Benzie and Strain FRAP method (19). The antioxidants in the plasma reduce a colorless ferric tripyridyltriazine complex (Sigma-Aldrich, St. Louis, MO, USA) to a blue ferrous complex that can be determined by comparing the absorbance change at 593 nm in test reaction mixture.

*Statistical analysis*

Data were analyzed using SPSS 20 (SPSS, Chicago, IL, USA). Descriptive analysis was operated for demographic and clinical data. All quantitative variables were tested for the normality by means of the Shapiro-Wilk test before analysis. Between groups comparisons were analyzed by Student›s t-test (for variables with normal distribution) and Mann-Whitney U test (for variables without normal distribution). The data of variables with normal distribution are expressed as means ± standard error of the means (SEM), and data of variables without normal distribution are expressed as median (interquartile ranges) (IQR). Comparative CT method (Schmittgen and Livak (20)) was used for analysis of the gene expression. Firstly, we calculated 2^-ΔCT^ (relative expression) from the measured ΔCT by Real Time PCR. Pearson correlation coefficients (r) were used to explain the associations between investigated genes. P-value of less than 0.05 was considered statistically significant. 

## Results

*Demographics and clinical characteristics*

This study was performed on 10 CAD patients with the median ± IQR age of 51 ± 4 years and 10 healthy subjects with the age of 50 ± 2. The anthropometric and laboratory differences among participants are shown in Table 1. CAD patients had significantly higher blood pressures (p < 0.0001), TC (p = 0.016), LDL cholesterol (p = 0.04), TG (p = 0.002), as well as significantly lower HDL cholesterol (p = 0.005) compared to the healthy group. However, BMI and Waist Circumference were not significantly different between the groups.

*PBMC viability measurement by MTT assay*

**Figure 1:**
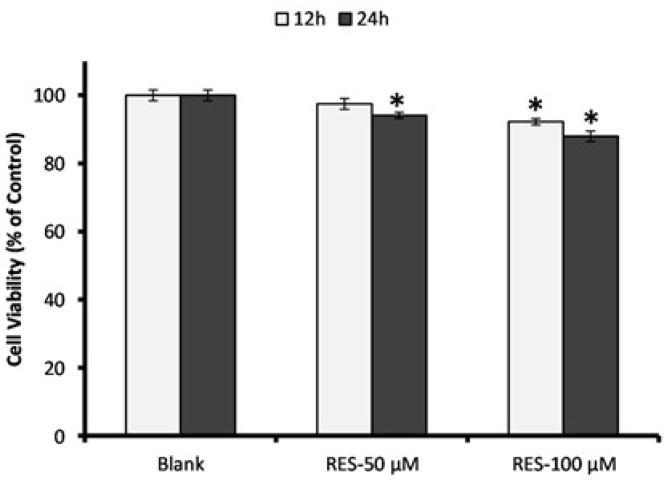
Assessment of PBMCs viability in response to resveratrol (RES) treatment**.** Cells were incubated with 50 and 100 µM RES for 12 h and 24 h, and cell viability was determined by MTT assay. All RES treatments, except RES 100 µM for 24 h, did not reduce the cell viability less than 90%. The MTT assay was performed in triplicate and data are reported from the mean of triplicates. Data are expressed as means ± SEM. * stands for p < 0.01.

Results of MTT assay for the effects of RES (50 and 100 µM) on PBMCs viability are shown in Figure 1. We observed no cytotoxicity effect after 12 h and 24 h exposure to the RES concentrations. Except 24 h incubation with 100 µM RES, the cell viability did not decrease less than 90% under exposure to the RES concentrations in comparison with blank group. After 12 h treatment with 50 µM RES, the cell viability was not significantly decreased compared to blank, thus we selected this concentration for further experiments.

*Influence of RES treatment on mRNA expression of β-catenin, MnSOD, and PPAR-δ*

**Figure 2:**
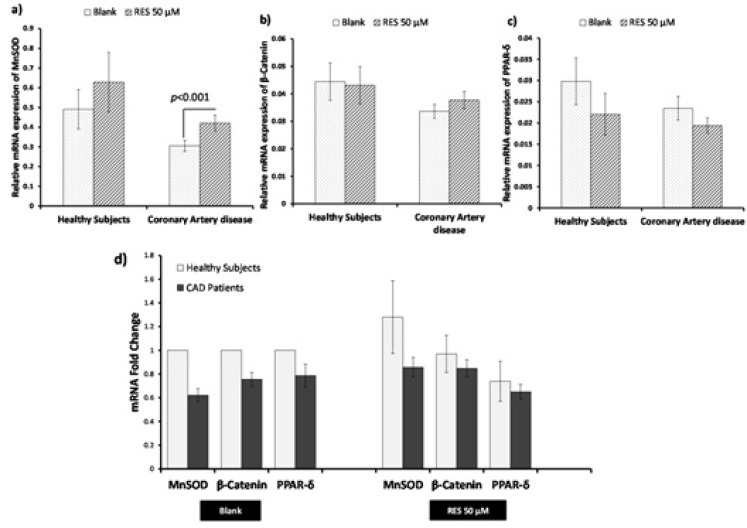
Effects of resveratrol (RES) on β-catenin, MnSOD, and PPAR-δ gene expression by real-time PCR. PBMCs were treated with 50 µM RES for 12 h incubation. a) A non-significant change in MnSOD mRNA was observed in healthy subjects after treatment with RES as compared to blank. Treatment with RES could significantly increase the mRNA level of MnSOD in CAD patients compared to blank. b) No significant differences were observed for β-catenin mRNA in healthy subjects and CAD patients after treatment with RES compared to their blank. c) No significant differences in PPAR-δ mRNA were observed for both healthy subjects and CAD patients after treatment with RES compared to their blanks. d) Between-group analysis showed non-significant higher levels of MnSOD, β-catenin and PPAR-δ in healthy subjects than CAD patients in both blank and RES treatment. RES was dissolved in DMSO, and blank groups (untreated cells) were containing only DMSO. In both blank and RES treated cells, DMSO was present at equal concentration (0.025%). All gene expression tests were performed in triplicate in each experiment, and the mean of duplicates were used for statistical analyses. Data are expressed as means ± SEM.

**Figure 3:**
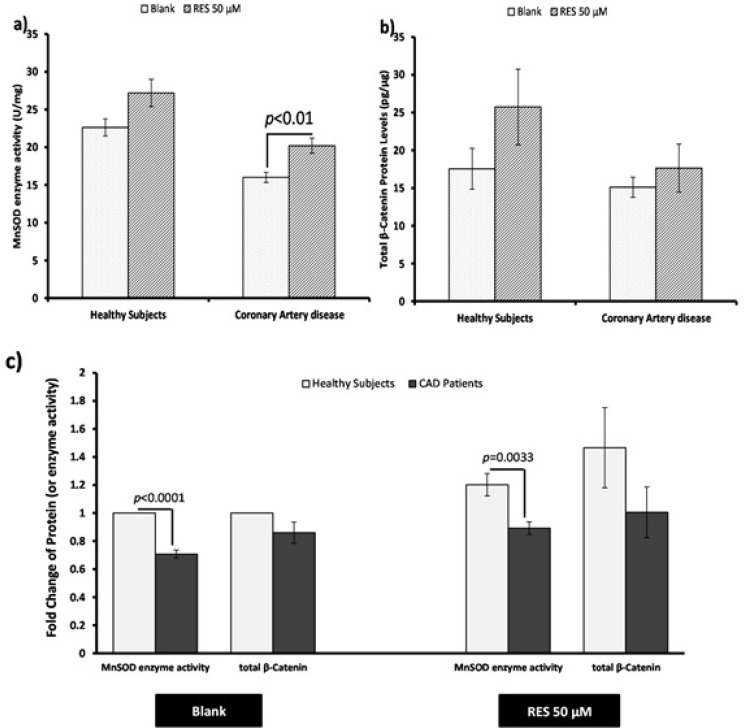
Pearson correlation coefficients of mRNA expression of genes (β-catenin, MnSOD and PPAR-δ). a) In blank groups; β-catenin and MnSOD mRNA expressions were significantly correlated in healthy subjects, while they were not significantly correlated in CAD patients. b) β-catenin and PPAR-δ mRNA showed significant positive correlations in both healthy subjects and CAD patients in blank groups. c) After treatment with resveratrol (RES) a significant positive correlation was found between β-catenin and MnSOD mRNA expressions in healthy subjects, but it was not observed in CAD patients. d) Also β-catenin and PPAR-δ mRNA showed significant positive correlations in both healthy subjects and CAD patients after treatment with RES. RES was dissolved in DMSO, and blank groups (untreated cells) were containing only DMSO. In both blank and RES treated cells, DMSO was present at equal concentration (0.025%).

The effects of RES on mRNA expression of the genes are shown in Figure 2. RES could not significantly increase the mRNA expression of MnSOD, β-catenin, and PPAR-δ in healthy subjects. However, RES could significantly increase the mRNA level of MnSOD in CAD patients compared to blank (Figure 2a; 38%, p < 0.0001). Moreover, no significant changes were observed for β-catenin and PPAR-δ mRNA levels of CAD patients after RES treatment (Figure 2b, 2c). Between groups analysis (Figure 2d) reveals no significant differences for β-catenin, MnSOD, and PPAR-δ mRNA expressions between healthy and CAD subjects in blank groups. Interestingly within group analysis showed that increased fold-change of MnSOD gene expression of CAD patients in response to RES is greater than healthy subjects, however, the MnSOD mRNA expression of CAD patients is non-significantly lower than healthy subjects (Figure 2d. fold-change: 1.49, p = 0.15). Between-group analysis revealed non-significant higher levels of β-catenin and PPAR-δ mRNA expressions in healthy subjects than CAD patients at both blank and RES treatments (Figure 2d.). The results of Pearson correlation coefficients of mRNA expression for target genes of canonical β-Catenin/Wnt and FOXO pathways are displayed in Figure 3. We observed a significant positive correlation between β-catenin and MnSOD mRNA expressions in healthy subjects (Figure 3a. r = 0.711, p < 0.0001); however, they were not significantly correlated in CAD patients (Figure 3a. r = 0.227; p = 0.16). Also β-catenin and PPAR-δ mRNA showed significant positive correlations in both healthy subjects (Figure 3b. r = 0.802; p < 0.0001) and CAD patients (Figure 3b. r = 0.801, p < 0.0001). RES-treated PBMCs of healthy subjects showed a significant positive correlation between β-catenin and MnSOD mRNA expressions (Figure 3c. r = 0.826, p < 0.0001), but it was not observed in CAD patients (Figure 3c. r = 0.401, p = 0.08). Moreover, significant positive correlations were found between β-catenin and PPAR-δ mRNA expressions of healthy subjects (Figure 3d. r = 0.578; p = 0.008) and CAD patients (Figure 3d. r = 0.641, p = 0.002) after treatment with RES.

*Measurement of MnSOD enzyme activity and total β-catenin protein*

**Figure 4:**
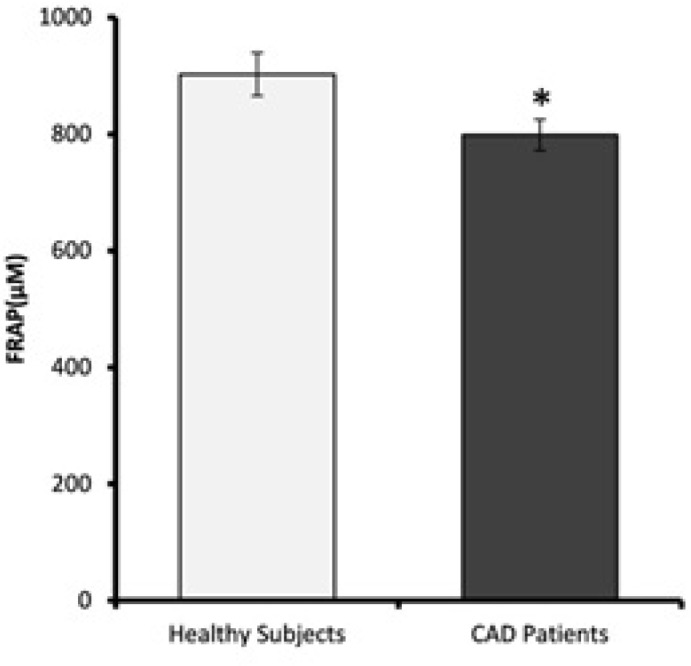
Effects of resveratrol (RES) on MnSOD enzyme activity and total β-catenin protein. PBMCs were treated with 50 µM RES for 12 h incubation. a) MnSOD enzyme activity was non-significantly increased in healthy subjects after treatment with RES. Also it was significantly increased in CAD patients after treatment with RES in comparison with blank. b) After treatment with RES a non-significant increasing trend was found for total β-catenin protein of healthy subjects compared to blank. Total β-catenin protein of CAD patients did not significantly increase after treatment with RES. c) Between-group differences showed higher MnSOD enzyme activitiy and total β-catenin protein levels for healthy subjects in comparison with CAD patients. RES was dissolved in DMSO, and blank groups (untreated cells) were containing only DMSO. In both blank and RES treated cells, DMSO was present at equal concentration (0.025%). All experiments were performed in duplicate, and the mean of duplicates were used for statistical analyses. Data are expressed as means ± SEM

**Figure 5:**
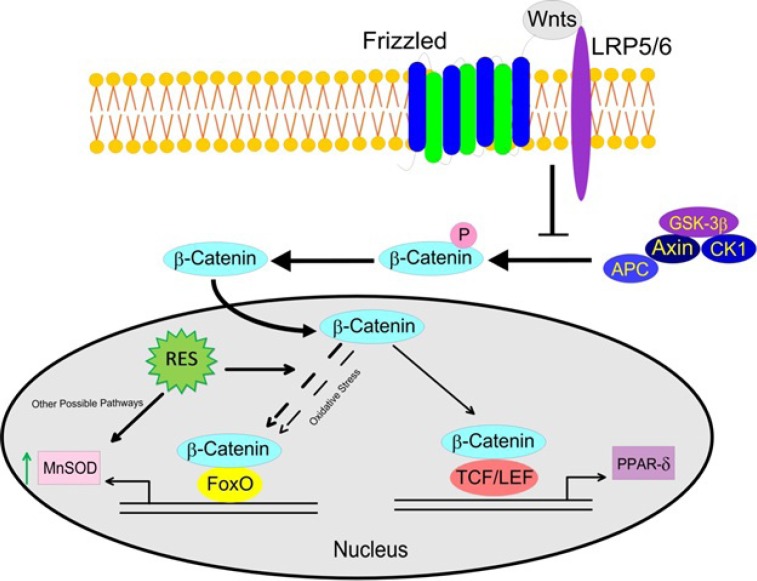
Pearson correlation coefficients of total β-catenin protein and MnSOD enzyme activity. a) In blank group; total β-catenin protein and MnSOD enzyme activity were significantly correlated in healthy subjects, while they were not significantly correlated in CAD patients. b) After treatment with resveratrol (RES), total β-catenin protein and MnSOD enzyme activity showed a significant positive correlation in healthy subjects, while no significant correlation was observed in CAD patients. RES was dissolved in DMSO, and blank groups (untreated cells) were containing only DMSO. In both blank and RES treated cells, DMSO was present at equal concentration (0.025%)

The effect of RES on total β-catenin protein level and MnSOD enzyme activity in PBMCs of both healthy subjects and CAD patients are shown in Figure 4. RES could non-significantly increase the MnSOD enzyme activity in healthy subjects (Figure 4a. 20%, p = 0.051). Also RES significantly increased the MnSOD enzyme activity in CAD patients compared to the blank (Figure 4a. 26%, p < 0.01). RES could not significantly change the total β-catenin protein level in CAD patients compared to blank (Figure 4b. 17%, p = 0.41). However, it could increase the total β-catenin protein in healthy subjects through a non-significant trend compared to blank (Figure 4b. 47%; p = 0.17). In addition, between groups analysis (Figure 4c.) showed that the MnSOD enzyme activity of healthy subjects was significantly higher than CAD patients (fold change = 1.41; p < 0.0001). More interestingly, while within group analysis showed a greater response to RES for MnSOD enzyme activity of CAD patients, it could not reach to the level of healthy subjects (Figure 4c. fold change = 1.35; p = 0.0033). Total β-catenin protein level of healthy subjects was non-significantly higher than CAD patients in both blank (Figure 4c. fold change = 1.16) and RES treatments (Figure 4c. fold change = 1.46). The results of correlation showed that total β-catenin protein and the MnSOD enzyme activity were significantly correlated in PBMCs of healthy subjects (Figure 5a; r = 0.446, p = 0.004), while they were not significantly correlated in CAD patients (Figure 5a; r = 0.019; p = 0.91). We observed that after treatment with RES, total β-catenin protein and MnSOD enzyme activity were significantly correlated in healthy subjects (Figure 5b. r = 0.535, p = 0.015), but it was not found in CAD patients (Figure 5b. r = 0.424; p = 0.063).

*FRAP values *

**Figure 6:**
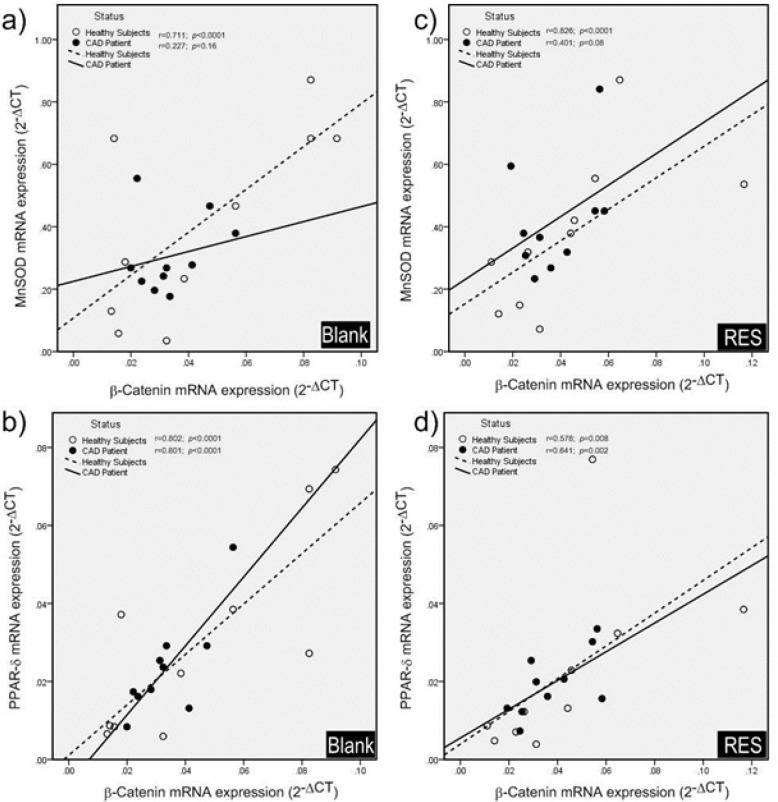
Plasma ferric reducing antioxidant power (FRAP) values. FRAP levels of CAD patients are significantly lower than healthy subjects. Data are expressed as means ± SEM. * p = 0.02

Plasma antioxidant levels of CAD patients and healthy subjects are shown in Figure 6. FRAP values of CAD patients were significantly lower than the healthy subjects (p = 0.02).

## Discussion

Oxidative stress is demonstrated as one of the paramount leading causes of atherosclerosis and consequent CAD (21). RES is one of the most extensively studied polyphenols with possible cardioprotective activities (11-13). However, its exact beneficial effects on signaling pathways involved in development of CAD remains to be known. To our knowledge, the dysfunctional canonical β-catenin/Wnt signaling might have a role in the pathogenesis of several metabolic disorders particularly CAD (6, 8, 15). β-catenin is a key mediator of the Wnt signaling pathway and acts as a co-activator for FOXO transcription factors under oxidative stress in order to up-regulate the free radical scavenging enzymes such as MnSOD (6, 22). On the other hand PPAR-δ is believed to be as a downstream target of the β-catenin/Wnt pathway (23), which conducts several favorable effects including suppressing atherogenic inflammation, decreasing insulin resistance, improving glycemic control, elevating high-density lipoprotein, and increasing fatty acid oxidation (23-25). 

**Figure 7:**
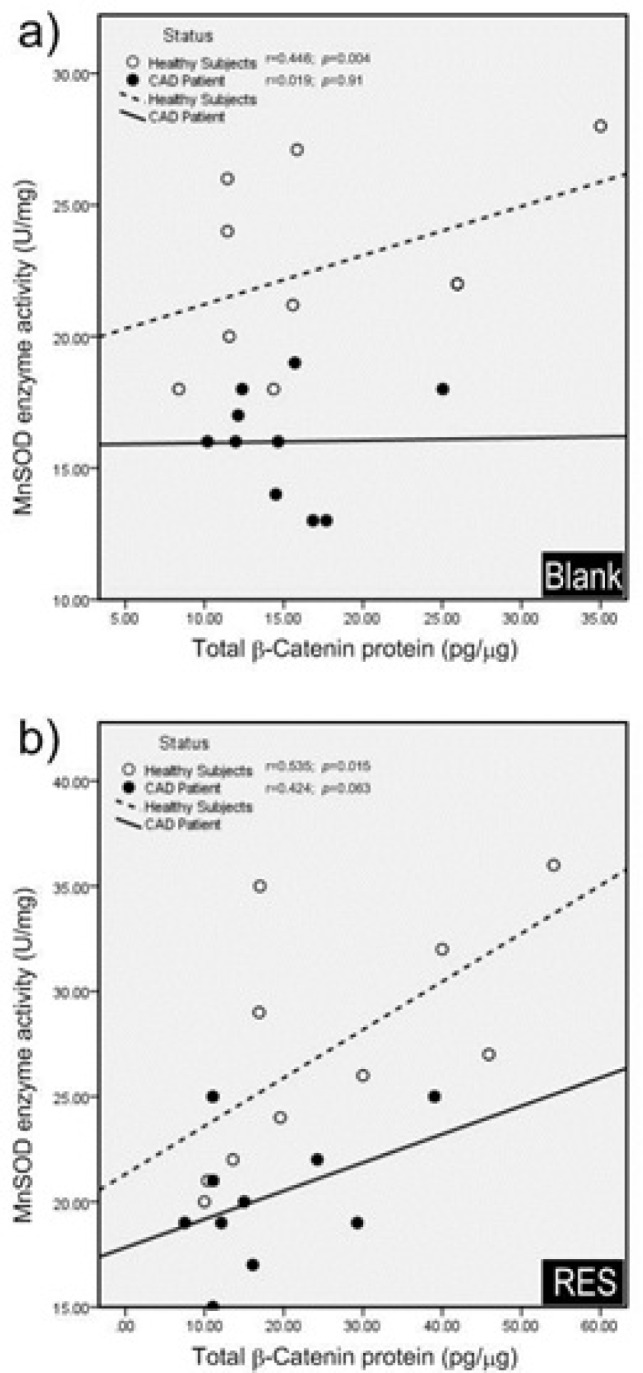
Effects of resveratrol (RES) on β-Catenin/Wnt and FOXO signaling pathways in PBMCs of CAD patients. The disrupted β-Catenin/FOXO pathway in CAD patients might be due to prolonged exposure to oxidative stress, which exerts a pathogenic role in CAD. RES could increase the MnSOD enzyme activity, however it was not through the β-Catenin/FOXO pathway. The β-catenin/Wnt pathway was intact in CAD patients but was not provoked by RES. (LRP5/6, Low density lipoprotein receptor-related protein 5/6; APC, Adenomatosis polyposis coli; GSK-3β, glycogen synthase kinase-3β; CK1, Casein kinase 1; TCF/LEF, T-cell factor/lymphoid enhancer factor; FOXO, Forkhead box O; MnSOD, Manganese superoxide dismutase; PPAR-δ, Proxisome prolifrator-activated receptor delta

This study was performed on PBMCs of three-vessel CAD patients with metabolic syndrome to assess the alterations of canonical β-catenin/Wnt and FOXO pathways in basal condition and in response to RES treatment. The main findings of our study are explained here: For the first time we demonstrated the impaired cross-talk between β-catenin/Wnt and FOXO pathways in PBMCs of CAD patients. Moreover, while RES could mildly improve the β-catenin/FOXO pathway in CAD patients, it could not repair this disrupted pathway. The β-catenin/Wnt pathway was intact in CAD patients but was not augmented by RES. Interestingly the MnSOD activity was significantly increased after treatment with RES independent from β-catenin/FOXO in CAD patients. The schematic representation of this impaired β-catenin /FOXO pathway and RES effects in CAD patients are displayed in Figure 7.

In basal condition both β-catenin/Wnt and FOXO pathways were intact in healthy subjects. In CAD patients, while the β-catenin/Wnt pathway was intact, the β-catenin/FOXO pathway was disrupted both at the mRNA and protein (or enzyme activity) levels. Total β-catenin protein and MnSOD enzyme activity of CAD patients appear to be lower than the healthy subjects. Evidence supports the fact that healthy subjects endure mild levels of oxidative stress (26, 27) which can lead to activation of FOXOs by Jun N-terminal kinases (JNK) phosphorylation (28) resulting in increased interactions between FOXO and β-catenin protein that up-regulates MnSOD enzyme activity (15, 27). This is in line with our finding regarding significantly increased MnSOD enzyme activity in healthy subjects compared to CAD patients. However, in CAD patients the prolonged exposure to moderate to high levels of oxidative stress might desensitize the FOXOs through acetylation and/or ubiquitination (28), which can disrupt the β-catenin/FOXO pathway at both mRNA and protein levels. In addition, the post-translational changes of MnSOD enzyme activity during chronic oxidative stress (29) in CAD patients might be the other cause of observed significant lower levels of MnSOD enzyme activity. The meta-analysis of Flores-Mateo *et al*. (3) agrees with our findings so that there was a significant inverse association between CAD and the levels of SOD activity.

Moreover, the fact that the number of involved coronary vessels of CAD patients is inversely associated with total antioxidant capacity (30); which might confirm the observation in our three-vessel CAD patients which showed significantly lower MnSOD enzyme activity and total plasma antioxidant power (in FRAP assay) compared to healthy subjects. Nevertheless, a study on diabetic patients (31) revealed that MnSOD enzyme activity was significantly increased in PBMCs of type 2 diabetic patients in comparison with healthy controls. The patients’ background of this study is different from our study so that only three CAD patients (30%) were included and six patients (60%) were under insulin treatment protocol, while we recruited a more homogenous sample of CAD patients (three-vessel CAD accompanying with metabolic syndrome history but with controlled hypertension and blood glucose). We believe the discrepancies in findings of oxidative and defensive profile of some studies might be the different inclusion and exclusion criteria used in these studies.

The slightly increased correlation between total β-catenin protein and MnSOD enzyme activity after treatment with RES reveals a modest augmentation of β-catenin/FOXO pathway in healthy subjects. The modest augmentation of β-catenin/FOXO pathway in our study might be justified by the findings of the other studies (32, 33) which support the fact that RES increases the phosphorylation of glycogen synthase kinase 3β (GSK-3β) which results in its inactivation. The GSK-3β inactivation leads to increase in total β-catenin protein and its consequent nuclear localization leading to increase in MnSOD enzyme activity (32, 33).

It was of particular importance that RES could not repair the disrupted β-catenin/FOXO pathway in CAD patients, while it could slightly improve this pathway. Also the MnSOD enzyme activity was significantly increased independent of total β-catenin protein level. This finding explains that indirect antioxidant effects of RES (increased activity of phase II enzymes *e.g*. MnSOD) in CAD patients might be regulated through protective signaling pathways other than β-catenin/FOXO pathway; *e.g*. NAD-dependent deacetylase sirtuin-1(SIRT1) /FOXO3a or Nuclear factor (erythroid-derived 2)-like 2 (Nrf2) transcription factor activation (34, 35). More exactly, the study of Robb *et al.* (34) the increased MnSOD expression and activity in MRC-5 cells induced by RES, might be due to the fact that RES stimulates migration of FOXO transcription factors to the nucleus (34, 36). In fact, RES has been shown to increase the sirtuin-catalyzed deacetylation of FOXO3a leading to its transcriptional activity and increasing the expression of MnSOD (34, 36). Moreover, evidence showed that RES increases Nrf2 transcription factor in primary rat hepatocytes (35), which is an important transcription factor for the expression of phase II anti-oxidant enzymes such as MnSOD and catalase (27, 35).

We observed that β-catenin/Wnt pathway in PBMCs of healthy subjects remains intact after treatment with RES. Evidence regarding the alterations in β-catenin/Wnt after treatment with RES in normal cell lines is controversial. Zhou *et al*. found that RES 50 µM augments the canonical β-catenin/Wnt signaling pathway in murine pluripotent mesenchymal cell line ST2 through increase in total β-catenin protein by inactivation of GSK-3β (33). Nevertheless, Hope *et al*. (37) demonstrated significant attenuation of Wnt pathway after treatment with low concentrations of RES which was accompanied with non-significant changes in β-catenin quantity in normal mucosa-derived cell line (NCM460). Preserved β-catenin/Wnt pathway in RES- treated PBMCs of healthy subjects in this study might be due to the fact that slight increases in total β-catenin protein could maintain the β-catenin/Wnt pathway, which can be indicated from the significant positive correlation of β-catenin and PPAR-δ after treatment with RES. To the best of our knowledge, we cannot provide certain explanations for these different effects of RES on canonical β-catenin/Wnt pathway in different studies; however, it can be attributed to the different cell lines, background of participants, and incubation time. It was astonishing that similar to healthy subjects, the correlation between β-catenin and PPAR-δ remained intact after RES treatment in CAD patients; however, RES could not reinforce the β-catenin/Wnt pathway in CAD patients and this might be due the fact that RES was unable to increase the total β-catenin protein. In support of our findings, Mani *et al*. (8) reported a missense mutation in Low density lipoprotein receptor-related protein 6 (LRP6), a co-receptor for the Wnt signaling pathway, leading to impaired Wnt signaling pathway in a family with early coronary artery disease. It indicates that observed irresponsiveness of β-catenin/Wnt pathway to RES might be due to the probable variations in Wnt signaling genes involved in CAD pathogenesis.

It should be also noticed that RES-induced stabilization of β-catenin in Wnt pathway depends on the cell line characteristics, incubation period, and participants’ background. For instance, in colon cancer cell lines (37) it has been demonstrated that RES reduces the expression of regulators of β-catenin localization which leads to the reduced β-catenin protein content that attenuates the Wnt pathway even in presence of Wnt3a (Wnt pathway activator). This attenuation of Wnt pathway reflects the anti-proliferative effect of RES in colon cancer cells which are shown to have mutations in Wnt pathway genes leading to its activation (37). Also interestingly RES is shown to attenuate the Wnt signaling pathway in a different manner in P19 colon cancer cell lines (38). So that RES was found to disrupt β-catenin/TCF complex, while did not affect the GSK-3 β inactivation and nuclear localization of β-catenin. However in murine pluripotent mesenchymal cell line ST2 (33) RES could augment the Wnt pathway through GSK-3β inactivation and the consequent increased levels of β-catenin protein (33). Thus it can be concluded that RES affects the β-catenin/Wnt pathway in PBMCs and other cell lines through distinct manners. 

Our study had some limitations. A relatively small sample size could considered as a major limitation in current study, however the results of this study based on the experiments on human cells of healthy subjects and three-vessel CAD patients supported by clinical and paraclinical information makes this study valuable as a ground for further investigations. In addition, given that we required a large amount of blood in order to isolate more PBMC from participants, we could not comprehensively explore the other genes involved in these two pathways.

Although we investigated a limited number of genes; we chose β-catenin as the pivotal regulator of both FOXO and Wnt pathways, and also we selected MnSOD and PPAR-δ as the target genes of these two pathways. Thus we suggest that other involved genes in these pathways require to be investigated to elucidate them in PBMCs of CAD patients. Since the cardioprotective effects of RES through other less-defined molecular mechanisms are remained to be known, RES administration as a therapeutic agent in cardiovascular diseases depends on further experimental studies.

**Table 1:** Anthropometric and laboratory differences between healthy subjects and coronary artery disease (CAD) patients.

	**Healthy Subjects** **N = 10**	**CAD Patients** **N = 10**	**p-value**
Characteristics	Median ± IQR	Median ± IQR	Mann-Whitney test
Age (years)	50 ± 2	51 ± 4	0.12
Body Mass Index (Kg/m^2^)	25.1 ± 2.20	24.95 ± 1.80	0.249
Waist Circumference (cm)	78.5 ± 9	80.5 ± 13	0.368
Total Cholesterol (mg/dL)	173 ± 7	188 ± 17	0.016
HDL Cholesterol (mg/dL)	40.5 ± 16	33 ± 16	0.005
LDL Cholesterol (mg/dL)	92 ± 5	98 ± 18	0.04
Triglyceride (mg/dL)	136 ± 31	166 ± 44	0.002
Systolic Blood Pressure (mmHg)	120 ± 5	140 ± 15	<0.001
Diastolic Blood Pressure (mmHg)	80 ± 0	90 ± 5	<0.001
Fasting Blood Sugar (mg/dL)	88.5 ± 12	93.5 ± 4.40	0.03

HDL, High density lipoprotein; LDL, Low density lipoprotein

Data are expressed as Median ± Interquartile range (IQR)

## Conclusion

Our data presented in this study demonstrate that the cross-talk between β-catenin/Wnt and FOXO pathways is impaired in CAD patients. Also the inefficient cellular defense mechanism by β-catenin/FOXO pathway which was found by our study suggests a pathogenic role for CAD. However, the exact molecular details regarding these pathways in the pathogenesis of CAD patients with metabolic syndrome are still obscure. Although RES did modestly increase the MnSOD enzyme activity in CAD patients, it could not repair the disrupted β-catenin/FOXO pathway. It is of particular importance that RES could preserve the canonical β-catenin/Wnt signaling pathway in CAD patients, but it could not augment it. Although our results provide valuable evidence about the role of RES in the regulation of oxidative stress, further studies are needed to clarify the therapeutic role of RES in CAD patients with different disease severity.

*Abbreviations*

CAD: Coronary artery disease; PBMC: Peripheral blood mononuclear cell; FOXO: Forkhead box O; TCF/LEF: T-cell factor/lymphoid enhancer factor; RES: Resveratrol; MnSOD: Manganese superoxide dismutase; PPAR-δ: Peroxisome proliferator-activated receptor delta; HDL: High density lipoprotein; LDL: Low density lipoprotein; CABG: Coronary artery bypass graft; BMI: Body mass index; FBS: Fasting blood sugar; TC: Total cholesterol; TG: Triglyceride; DMSO: Dimethyl sulfoxide; MTT: 3-(4, 5-dimethylthiazol-2-yl)-2, 5 diphenyl tetrazolium bromide; KCN: Potassium cyanide; EIA: Enzyme immunoassay; FRAP: Plasma ferric reducing antioxidant power; JNK: Jun N-terminal kinases; GSK-3β: Glycogen synthase kinase 3β; SIRT1: NAD-dependent deacetylase sirtuin-1; Nrf2: Nuclear factor (erythroid-derived 2)-like 2; LRP5/6: Low density lipoprotein receptor-related protein 5/6; APC:Adenomatosis polyposis coli; CK1: Casein kinase 1.

*Authors*᾽* contributions*

MSB, AH, TG, RM, MB, MS and PP conceived of the study and participated in its design. All authors contributed writing and editing the manuscript. AH, MSB, and MB performed data interpretation and statistical analysis. MS helped to collect the samples. MSB, AH, TG, RM, MB and PP performed the experiments. All authors read and approved the final manuscript.
